# Global blood gene expression profiles following a breast cancer diagnosis—Clinical follow-up in the NOWAC post-genome cohort

**DOI:** 10.1371/journal.pone.0246650

**Published:** 2021-03-08

**Authors:** Karina Standahl Olsen, Marit Holden, Jean-Christophe Thalabard, Lill-Tove Rasmussen Busund, Eiliv Lund, Lars Holden

**Affiliations:** 1 UiT The Arctic University of Norway, Tromsø, Norway; 2 Norwegian Computing Center, Oslo, Norway; 3 MAP5, UMR CNRS 8145, Université Paris Descartes, USPC, Paris, France; 4 The University Hospital of North Norway, Tromsø, Norway; 5 The Cancer Registry of Norway, Oslo, Norway; CNR, ITALY

## Abstract

**Objective:**

This explorative study aimed to assess if there are any time-dependent blood gene expression changes during the first one to eight years after breast cancer diagnosis, which can be linked to the clinical outcome of the disease.

**Material and methods:**

A random distribution of follow-up time from breast cancer diagnosis till blood sampling was obtained by a nested, matched case-control design in the Norwegian Women and Cancer Post-genome Cohort. From 2002–5, women were invited to donate blood samples, regardless of any cancer diagnosis. At end of the study period in 2015, any cancer diagnoses in the 50 000 participants were obtained via linkage to the Norwegian Cancer Registry. For each breast cancer patient (n = 415), an age- and storage time-matched control was drawn. The design gave a uniform, random length of follow-up time, independent of cancer stage. Differences in blood gene expression between breast cancer cases and controls were identified using the Bioconductor R-package limma, using a moving window in time, to handle the varying time elapsed from diagnosis to blood sample.

**Results:**

The number of differentially expressed genes between cases and controls were close to 2,000 in the first year after diagnosis, but fell sharply the second year. During the next years, a transient second increase was observed, but only in women with metastatic disease who later died, both compared to invasive cases that survived (p<0,001) and to metastatic cases that survived (p = 0.024). Among the differentially expressed genes there was an overrepresentation of heme metabolism and T cell-related processes.

**Conclusion:**

This explorative analysis identified changing trajectories in the years after diagnosis, depending on clinical stage. Hypothetically, this could represent the escape of the metastatic cancer from the immune system.

## Introduction

Metastases are the major cause of death among breast cancer patients. Even though survival has increased over the last decades in countries like Norway [1], early diagnosis of metastases and prediction of survival should be improved. Blood gene expression profiles at diagnosis have been explored as a biomarker for breast cancer [2], and in addition, such profiles have been shown to discriminate between breast cancer patients and healthy women several years before diagnosis, when stratified by stage and detection method [3, 4]. However, to this day, neither gene expression nor methylation [5] can be used to diagnose this disease.

Changes in global gene expression after diagnosis among breast cancer patients compared to healthy women have not been carefully studied in a systems epidemiology design. Analyses of gene expression from immune cells in blood could potentially improve the understanding of the immune response during the carcinogenetic and metastatic processes of breast cancer in the years after diagnosis. In the present study, we used the Norwegian Women and Cancer (NOWAC) Post-genome Cohort [6] to explore global gene expression profiles from immune cells in blood in the first 8 years after breast cancer diagnosis, stratified by cancer stage and vital status (survival/death at end of follow-up). We introduced a novel design for clinical research with randomized length of follow-up time that gave a uniform distribution of the observed time independent of disease status.

## Results

**Fig 1:**
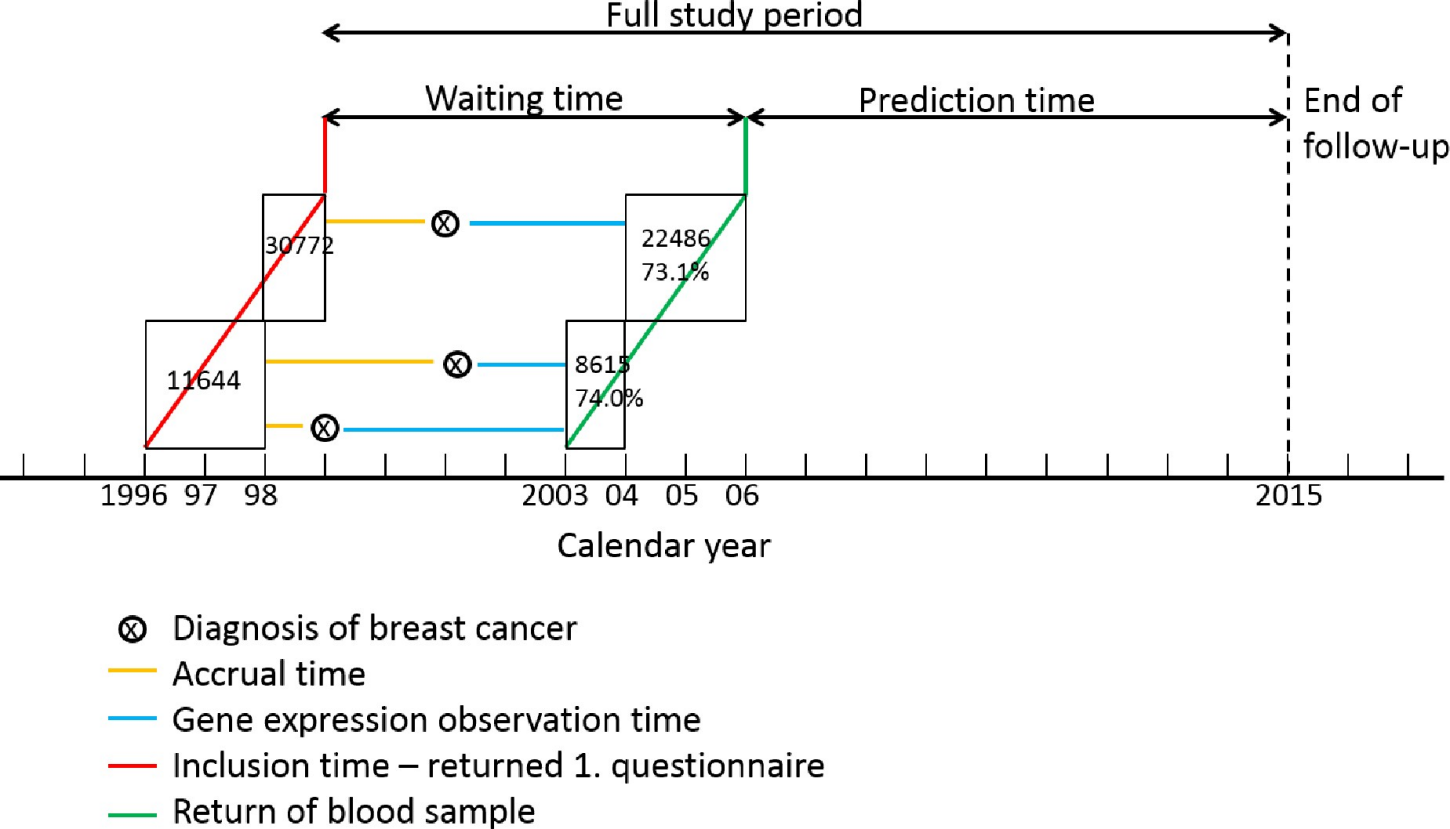
Design of the post-diagnostic study with definitions of the different observation times.

Of the 415 breast cancer cases included in this analysis, 51 had *in situ* breast cancer, 227 had invasive breast cancer, and 137 had metastatic breast cancer (Table 1). Tumor size was larger for cases with lymph node metastases than for those with invasive cancer (p<0.001). Among all cases, seven cases were diagnosed with metastasis or a second breast cancer: 1 case diagnosed less than one year after the primary diagnosis, 4 cases 1-<4 years after, and 2 cases were diagnosed more than 4 years after the primary diagnosis. Among the 227 invasive cases, six developed metastasis in the years up to 2018. There was a maximum of 8 years between cancer diagnosis and blood sampling. The study participants were sampled at random from the NOWAC study regardless of disease status, hence, the gene expression observation time was randomly distributed (Fig 1). The median time to death for the metastatic cases who later died was 8.16 years. Receptor status was insufficiently reported to the Cancer Registry in the years where our cases were diagnosed, but for breast cancer in Norway, the distribution is as follows: HER2+ 13%, ER+ 83%, PR+ 53%, triple negative 9,1%. We found no statistically significant differences between cases and controls in well-established risk factors of breast cancer: BMI, smoking status, and number of children (S1 Appendix).

**Table 1:** Overview of the study population (415 cases), according to breast cancer stage at the time of diagnosis.

		*In situ*	Invasive, no metastases	Regional metastasis to lymph nodes	Regional metastasis to muscles	Distant metastases	Sum
Year after diagnosis	1	7	34	11	0	2	54
	2	7	55	24	1	3	90
	3	11	38	22	0	1	72
	4	9	32	22	0	1	64
	5	4	17	13	0	1	35
	6	6	19	14	0	1	40
	7	6	24	19	0	0	49
	8	1	8	1	1	0	11
	Sum	51	227	126	2	9	415
Tumor size (cm)	0.3	0	12	2	0	1	15
	0.75	0	43	11	0	1	55
	1	0	1	0	0	0	1
	1.5	0	108	46	0	2	156
	3.5	0	28	40	0	2	70
	5	0	2	2	0	1	5
	Mean		1.58	2.26		2.29	

### Gene expression observation time

**Fig 2:**
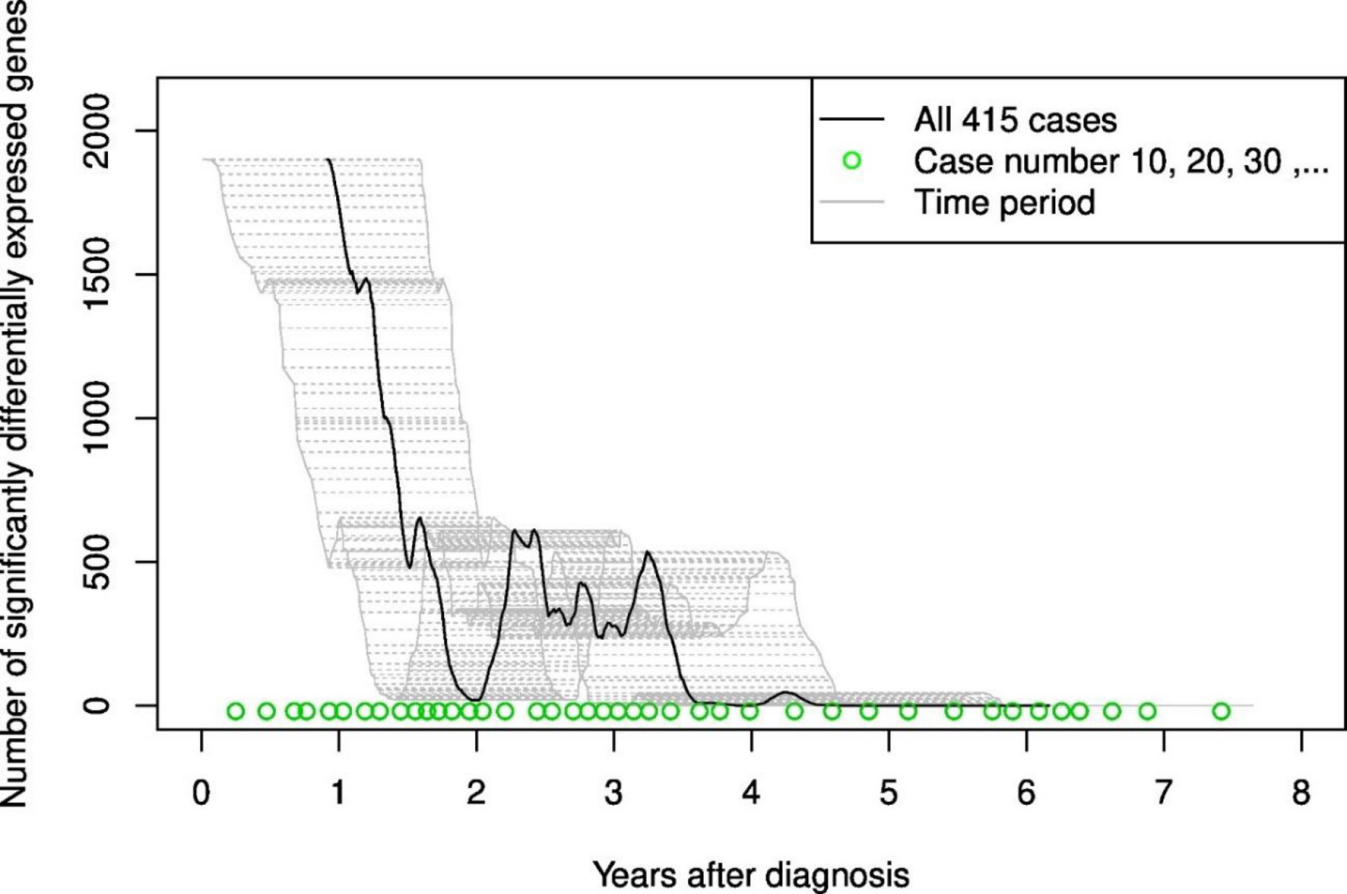
Number of significantly differentially expressed genes for each follow-up time period.

We performed limma analyses with a moving window in time to identify time periods after breast cancer diagnosis where cases and controls had different gene expression profiles (Fig 2). Looking at all 415 case-control pairs together, the number of differentially expressed genes was close to 2,000 in year 1, with a steep decline almost to zero in year 2. This was followed by a *second transient increase* up to around 500 differentially expressed genes between 24 and 36 months after breast cancer diagnosis, which are no longer present after 36 months. Merging all the time periods into one time period, the number of significantly differentially expressed genes was 2849 for all 415 cases, 0 for the 51 *in situ* cases, 532 for the 227 invasive cases, and 3310 for the 137 metastatic cases, respectively.

**Fig 3:**
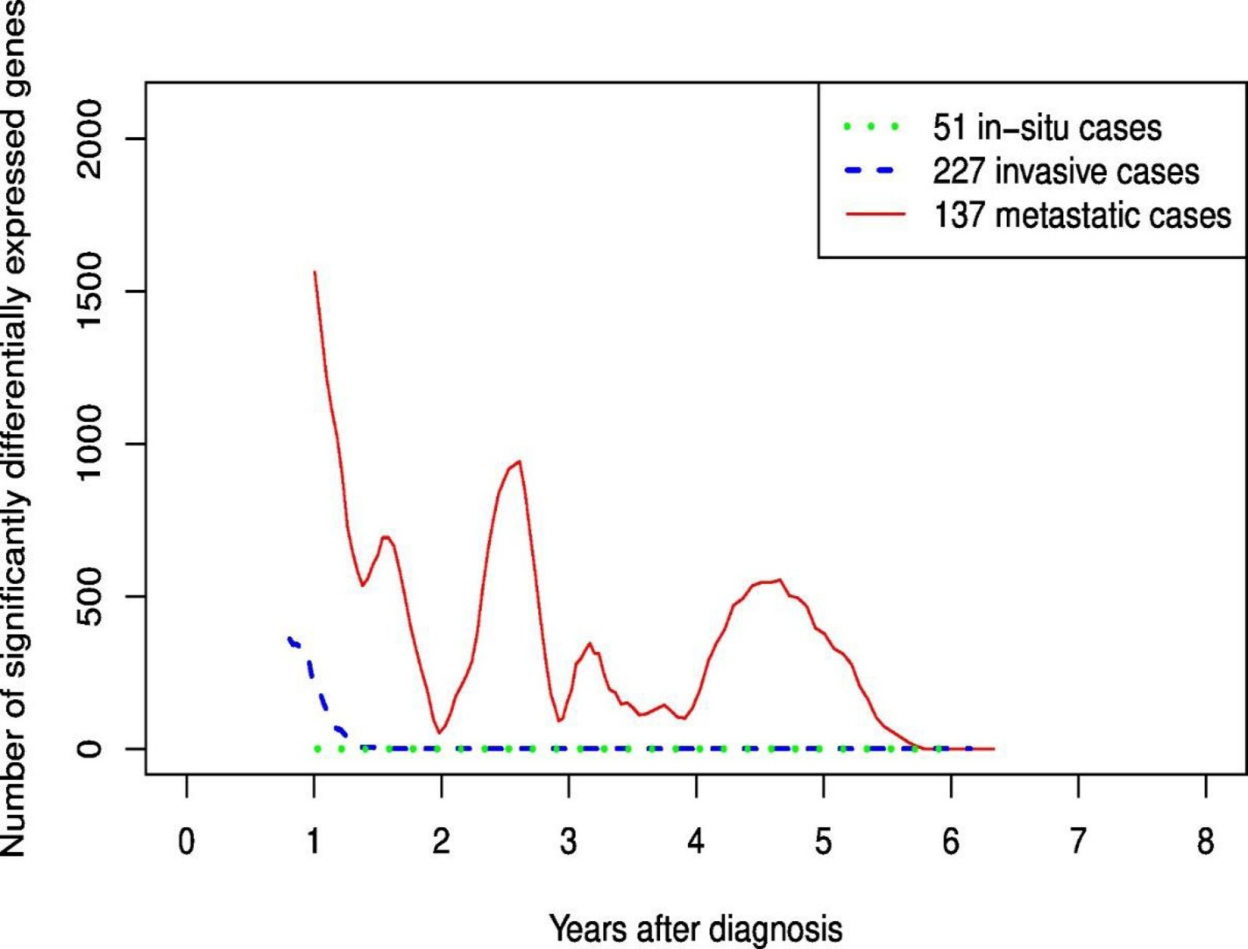
Number of significantly differentially expressed genes for each follow-up time period stratified on stage of breast cancer; invasive and metastatic.

The number of differentially expressed genes stratified by cancer stage (Fig 3) showed that almost all differentially expressed genes in the second transient increase were observed in the metastatic group; no differentially expressed genes were observed in *in situ* cases and only a few in invasive cases. When the second transient increase was defined as differentially expressed genes only after 18 months, the number of genes up-regulated/down-regulated were 819/1440 for metastatic and 2/7 for invasive cases.

**Fig 4:**
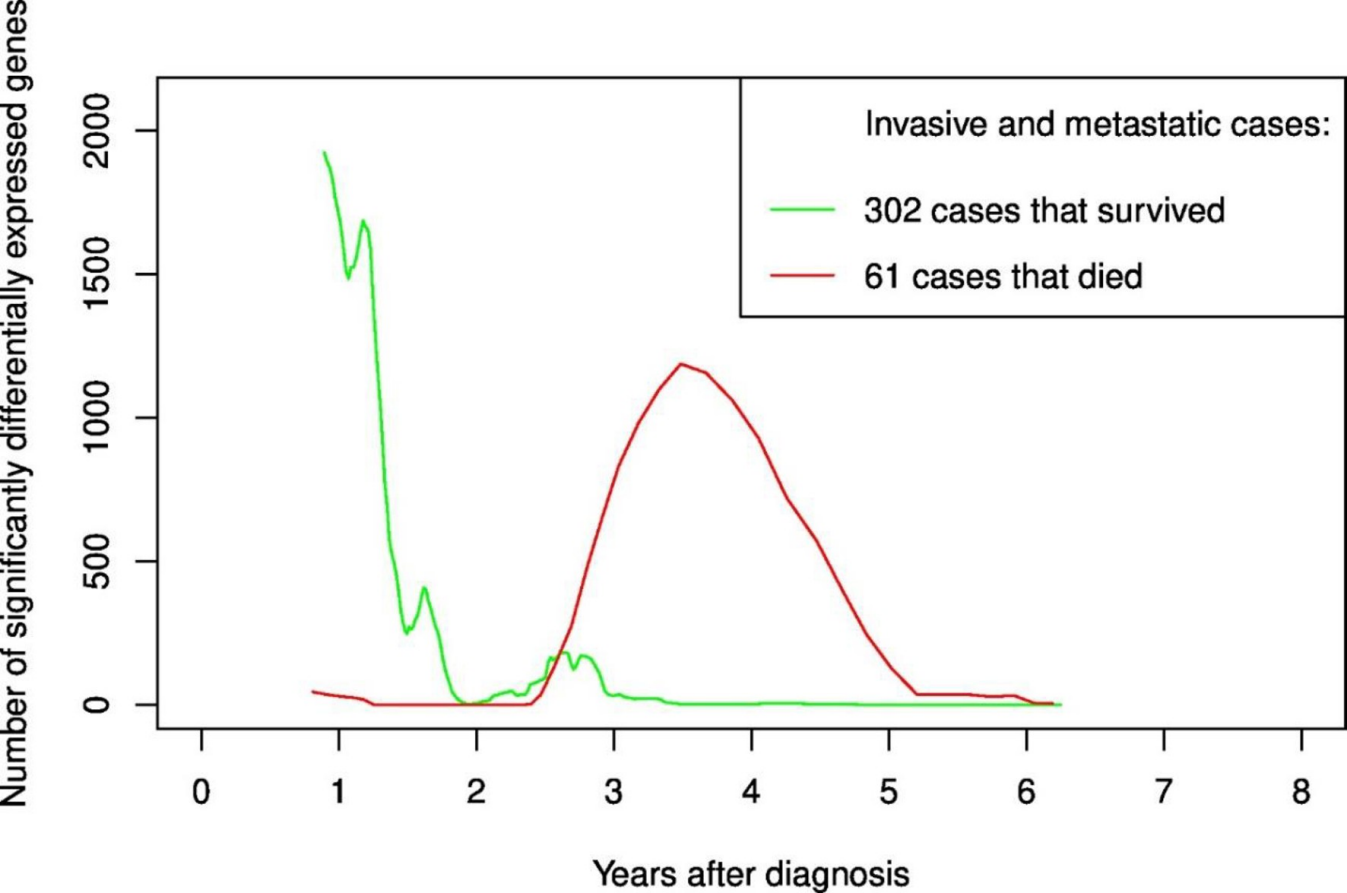
Number of significantly differentially expressed genes for each follow-up time period stratified on vital status at the end of follow-up; death or survival.

When stratified by vital status (survival/death, Fig 4), differentially expressed genes were almost exclusively found in cases who later died. Regardless of cancer stage, breast cancer cases who died showed no differentially expressed genes the first years after diagnosis, which was in contrast to invasive cases who survived. Using the same definition of the second transient increase (after 18 months), the number of genes up-/down-regulated were 998/1470 and 193/455 for breast cancer cases who were dead or alive, respectively, at end of follow-up.

### Differentially expressed genes 18 months after breast cancer diagnosis–a two-dimensional, stratified analysis of the second transient increase

**Fig 5:**
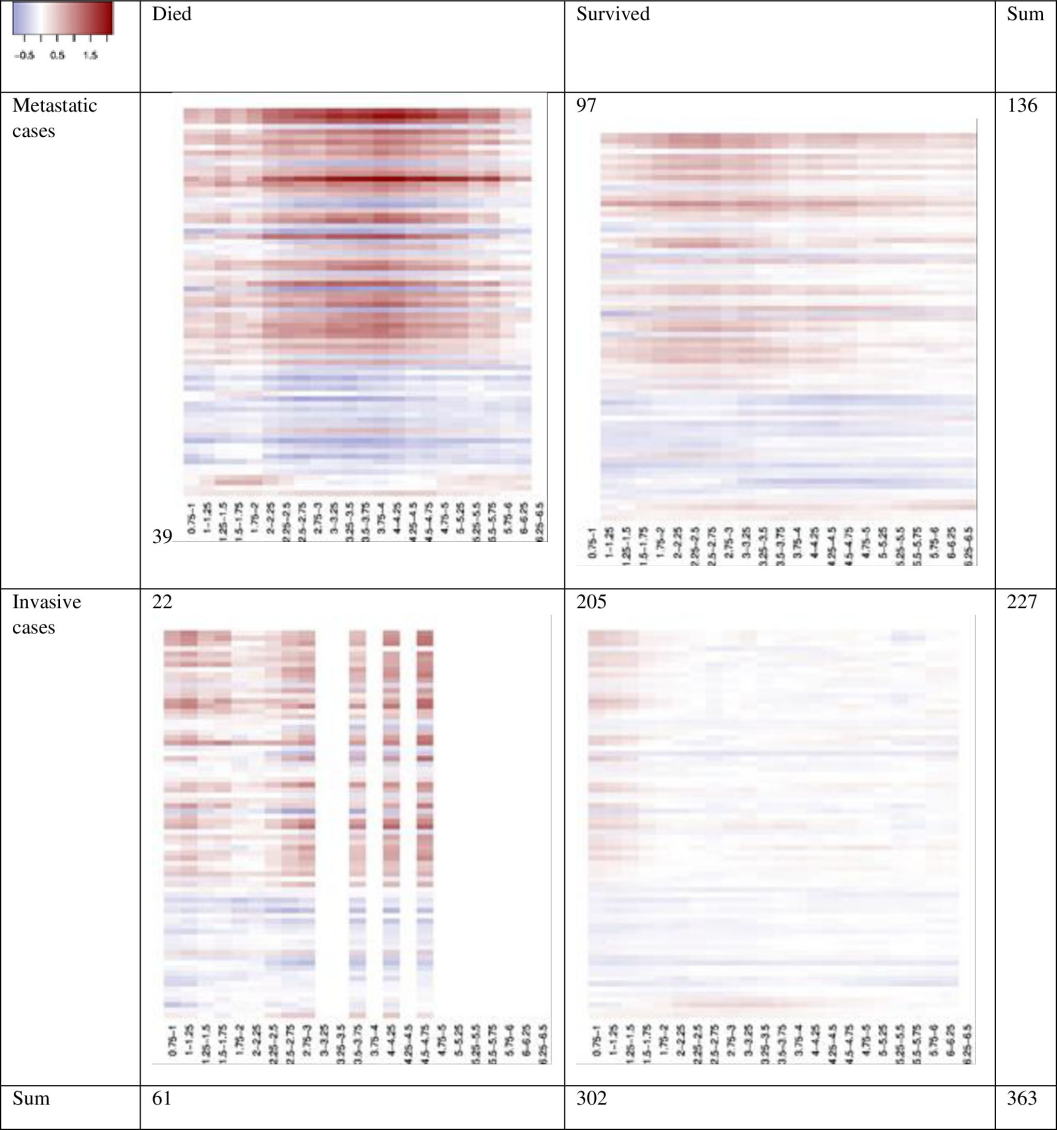
Heat maps with 74 selected genes in each stratum; invasive versus metastatic, and death or survival, at end of follow-up. The 50 topmost genes in the heat maps are the 50 top genes for the metastatic cases that died, the next 19 are the significantly up-regulated or down-regulated genes for the metastatic cases that survived, and the last five are the significantly up-regulated or down-regulated genes for the invasive cases that survived. There were no significantly up-regulated or down-regulated genes for the invasive cases that died. The heat maps show log fold change for each gene (y-axis) for each quarter of the years after diagnosis (x-axis).

The gene expression patterns over time were plotted as heat maps with fold change between cases and controls (Fig 5), based on a 2x2 stratification. Data are shown for genes that were significantly differentially expressed in at least one time period, and the figure only includes data from 18 months or more after breast cancer diagnosis. The figures gives the top 50 genes in the group of metastatic patients that died, and the 24 genes that were significantly differentially expressed in at least one of the other groups. Cancer stage was stratified on invasive or metastatic breast cancer, and vital status was stratified on death or survival at the end of follow-up. The number of cases in the stratified analyses of gene expression related to the second transient increase decreased by one-fifth due to the restriction of time periods.

We tested whether there were statistically significant differences in the numbers of significant genes when comparing the four strata against each other, using data from the blood samples taken at least 18 months after diagnosis (Table 2). We repeatedly sampled 30 of the 79 (153) cases, and found the number of up-regulated/down-regulated genes for each sampled dataset. Simulations (N = 1000) confirmed that the 648+979 = 1627 up-/down-regulated genes for the metastatic cases that died (gene list in S2 Appendix) are significantly larger than the 2+17 = 19 genes for the metstatic cases that survived (p = 0.024), and also significantly larger than the 4+1 = 5 genes for the invasive cases that survived (p = 0.001). A similar comparison between the invasive cases that died and the metastatic cases that died did not show a significant difference in the number of significant genes. As in this case we sampled only 16 of 30 cases, it is not suprising that that no significantly up-/down-regulated genes were found in many of the simualtions.

**Table 2:** Comparison of the number of differentially expressed genes between the four strata of breast cancer cases, using using metastatic breast cancer patients that died as reference.

	N cases at diagnosis	N cases >18 months after diagnosis	N genes up-/down-regulated	p-value
Metastatic cases that died	39	30	648/979	Ref.
Metastatic cases that survived	97	79	2/17	0.024
Invasive cases that died	22	16	0/0	ns
Invasive cases that survived	205	152	4/1	0.001

**Fig 6:**
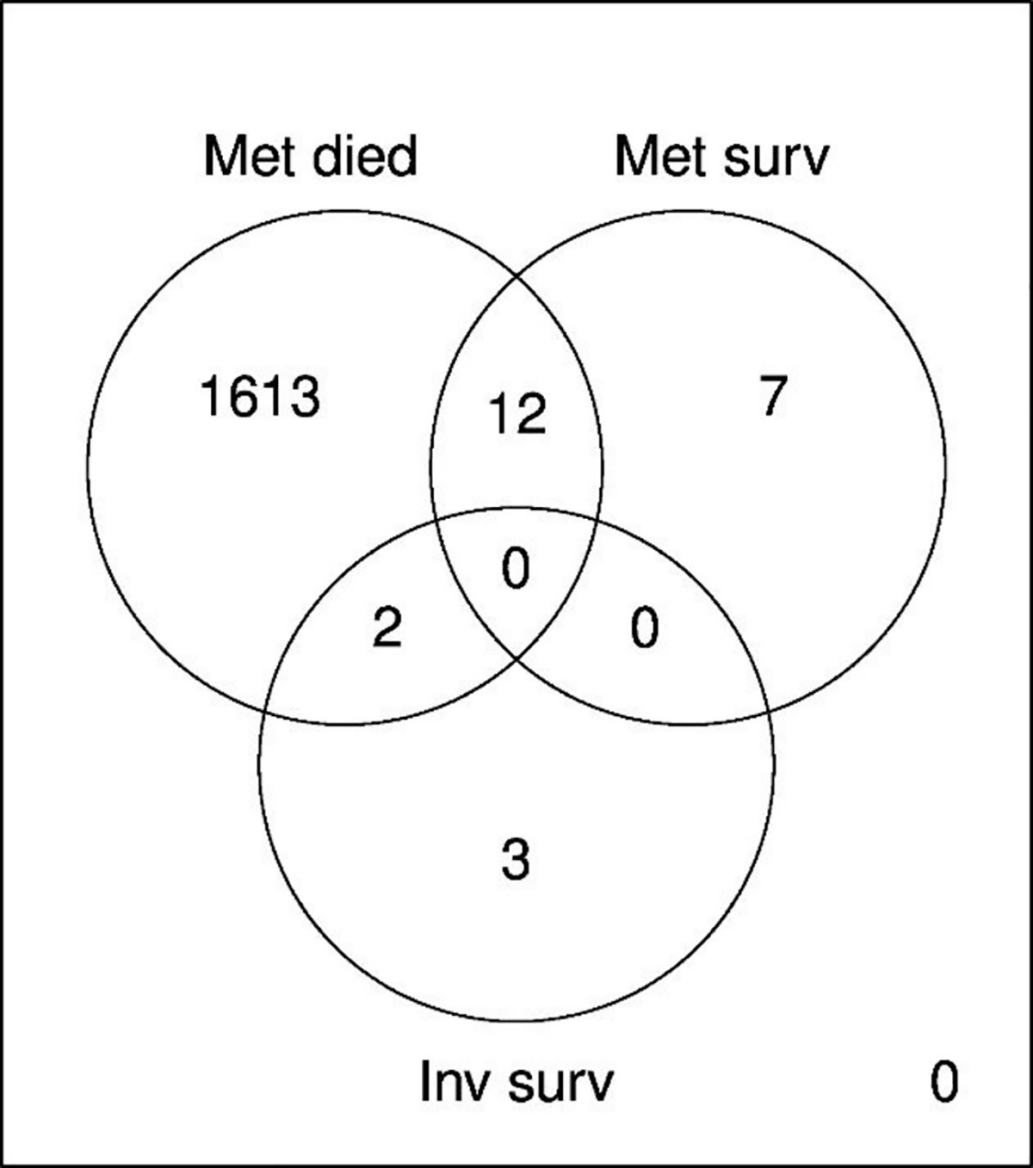
Venn diagram for the three strata with significantly different gene expression profiles; metastatic cases who died (labeled as met died), metastatic cases who survived (met surv), and invasive cases who survived (inv surv).

There was very little overlap between the groups when looking at all differentially expressed genes in the follow-up period (Fig 6), and no genes were significantly differentially expressed in all of the three groups.

To explore biological processes, we took two approaches. First we focused on the 1627 genes that were significantly differentially expressed in the metastatic cases who later died (S2 Appendix). The gene list was sorted by the number of time periods out of the total 22 periods after 18 months post diagnosis, where each gene was significant. Hence, the top genes are the ones that were significant in the highest number of time periods. A Fisher’s test revealed no significant gene sets at our chosen level of significance (FDR<5%) when comparing our full gene list to the hallmark and immunological gene sets in MSigDB. Focusing only on the top genes presented in the heatmap (Fig 5), 3 hallmark gene sets, and 17 immunological gene sets turned out significant in an overlap analysis at an FDR of 5% (Table 3). Out of the 17 immunological gene sets, 15 could be characterized as reflecting parts of either adaptive or innate immunity. Ten of these 15 gene sets reflected adaptive immunity, mainly aspects of T cell biology. The three significant hallmark gene sets were heme metabolism, apoptosis, and cholesterol metabolism.

**Table 3:** Significant gene sets (overlap analysis, FDR<5%) among the top differentially expressed genes, in blood gene expression profiles from metastatic cases who died during follow-up, compared to controls.

Gene set name	# Genes in Set (K)	# Genes in overlap (k)	k/K	p-value	FDR q-value	Description from MSigDB
hallmark_heme_metabolism	200	13	0.0650	4.17e-20	2.08e-18	Genes involved in metabolism of heme (a cofactor consisting of iron and porphyrin) and erythroblast differentiation.
hallmark_apoptosis	161	3	0.0186	7.33e-4	1.83e-2	Genes mediating programmed cell death (apoptosis) by activation of caspases.
hallmark_cholesterol_homeostasis	74	2	0.0270	2.98e-3	4.97e-2	Genes involved in cholesterol homeostasis.
gse34205_rsv_vs_flu_inf_infant_pbmc_up	192	14	0.0729	2.51e-22	1.22e-18	Genes up-regulated in comparison of peripheral blood mononuclear cells (PBMC) from infancts with acute RSV infection versus PBMCs from infants with acute influenza infection.
gse34205_healthy_vs_rsv_inf_infant_pbmc_dn	200	11	0.0550	2.59e-16	6.3e-13	Genes down-regulated in comparison of peripheral blood mononuclear cells (PBMC) from healthy donors versus PBMCs from infanct with acute RSV infection.
gse7852_treg_vs_tconv_thymus_up	200	6	0.0300	8.54e-8	1.39e-4	Genes up-regulated in comparison of thymus regulatory T cells versus thymus conventional T cells.
gse21546_sap1a_ko_vs_sap1a_ko_and_elk1_ko_anti_cd3_stim_dp_thymocytes_up	200	5	0.0250	2.68e-6	2.61e-3	Genes up-regulated in double positive thymocytes stimulated by anti-CD3: ELK4 knockout versus ELK1 and ELK4 knockout.
gse35685_cd34pos_cd38neg_vs_cd34pos_cd10neg_cd62lpos_bone_marrow_dn	200	5	0.0250	2.68e-6	2.61e-3	Genes down-regulated in the bone marrow CD34+ cells: CD38- versus MME- SELL+.
gse10856_ctrl_vs_tnfrsf6b_in_macrophage_up	186	4	0.0215	5.17e-5	1.96e-2	Genes up-regulated in comparison of macrophages treated with control (hIgG1) versus those treated with TNFRSF6B.
gse11057_eff_mem_vs_cent_mem_cd4_tcell_dn	188	4	0.0213	5.39e-5	1.96e-2	Genes down-regulated in comparison of effector memory T cells versus central memory T cells from peripheral blood mononuclear cells (PBMC).
gse19888_adenosine_a3r_act_vs_tcell_membranes_act_and_a3r_inh_pretreat_in_mast_cell_up	191	4	0.0209	5.73e-5	1.96e-2	Genes up-regulated in HMC-1 (mast leukemia) cells: Cl-IB-MECA versus incubated with the peptide ALL1 followed by stimulation with T cell membranes.
gse22501_peripheral_blood_vs_cord_blood_treg_up	197	4	0.0203	6.46e-5	1.96e-2	Genes up-regulated in T reg from: peripheral blood versus cord blood.
gse24634_naive_cd4_tcell_vs_day3_il4_conv_treg_up	198	4	0.0202	6.59e-5	1.96e-2	Genes up-regulated in comparison of naive T cells at day 0 versus CD25+ regulatory T cell (Treg) treated with IL4 at day 3.
gse25088_rosiglitazone_vs_il4_and_rosiglitazone_stim_macrophage_day10_up	199	4	0.0201	6.72e-5	1.96e-2	Genes up-regulated in wildtype bone marrow-derived macrophages treated with rosiglitazone: control versus IL4.
gse17186_memory_vs_naive_bcell_dn	200	4	0.0200	6.85e-5	1.96e-2	Genes down-regulated in B lymphocytes: memory versus naïve.
gse17721_ctrl_vs_cpg_6h_bmdc_up	200	4	0.0200	6.85e-5	1.96e-2	Genes up-regulated in comparison of control dendritic cells (DC) at 6 h versus those stimulated with CpG DNA (TLR9 agonist) at 6 h.
gse20366_treg_vs_tconv_dn	200	4	0.0200	6.85e-5	1.96e-2	Genes down-regulated in comparison of TregCD103-Klrg1 versus TconvLP.
gse25123_il4_vs_il4_and_rosiglitazone_stim_macrophage_day10_up	200	4	0.0200	6.85e-5	1.96e-2	Genes up-regulated in wildtype bone marrow-derived macrophages treated with IL4: control versus rosiglitazone.
gse2826_wt_vs_btk_ko_bcell_dn	200	4	0.0200	6.85e-5	1.96e-2	Genes down-regulated in comparison of primary splenic B cells from wild type mice versus those from BTK knockout mice.
gse41978_id2_ko_and_bim_ko_vs_bim_ko_klrg1_low_effector_cd8_tcell_up	200	4	0.0200	6.85e-5	1.96e-2	Genes up-regulated in KLRG1 low CD8 T effector cells during infection: ID2 and BCL2L11 versus BCL2L11 knockout.

In the second approach to biological processes, we used fgsea to identify significantly enriched gene sets (Table 4). The fgsea was based on a gene ranking that reflects the number of time periods with significant FDR q-values for each gene. At an FDR of 5%, 1 gene set from the hallmark collection was significant (heme metabolism), and 5 from the immunological gene set collection. Three out of the five significant immunological gene sets represent data from peripheral blood mononuclear cells (PBMCs). There are three gene sets that turn out significant in both gene set approaches (Fisher’s test and fgsea): heme metabolism from the hallmark collection, as well as gse11057_cd4_cent_mem_vs_pbmc_up and gse34205_healthy_vs_rsv_inf_infant_pbmc_dn from the immunological collection.

**Table 4:** Results from the fgsea analysis of enriched gene sets in blood gene expression profiles from metastatic cases who died during follow-up, compared to controls.

a) Enriched gene sets from the MSigDB H collection: hallmark gene sets				
	Gene Set Name	p-value	FDR q-value	
1	hallmark_heme_metabolism	1,00e-05	5,00e-04	Genes involved in metabolism of heme (a cofactor consisting of iron and porphyrin) and erythroblast differentiation.
2	hallmark_myc_targets_v2	2,10e-02	5,26e-01	A subgroup of genes regulated by MYC—version 2 (v2).
3	hallmark_tgf_beta_signaling	6,06e-02	9,33e-01	Genes up-regulated in response to TGFB1 [GeneID = 7040].
4	hallmark_unfolded_protein_response	8,88e-02	9,33e-01	Genes up-regulated during unfolded protein response, a cellular stress response related to the endoplasmic reticulum.
5	hallmark_uv_response_up	9,65e-02	9,33e-01	Genes up-regulated in response to ultraviolet (UV) radiation.
6	hallmark_myogenesis	1,12e-01	9,33e-01	Genes involved in development of skeletal muscle (myogenesis).
7	hallmark_myc_targets_v1	1,76e-01	1,00e+00	A subgroup of genes regulated by MYC—version 1 (v1).
8	hallmark_apoptosis	2,38e-01	1,00e+00	Genes mediating programmed cell death (apoptosis) by activation of caspases.
9	hallmark_notch_signaling	2,45e-01	1,00e+00	Genes up-regulated by activation of Notch signaling.
10	hallmark_dna_repair	2,51e-01	1,00e+00	Genes involved in DNA repair.
b) Enriched gene sets from the MSigDB C7 collection: immunologic signature gene sets				
	Gene Set Name	p-value	FDR q-value	Description from MSigDB
1	gse11057_cd4_cent_mem_vs_pbmc_up	1,00e-05	1,22e-02	Genes up-regulated in comparison of central memory T cells versus peripheral blood mononuclear cells (PBMC).
2	gse34205_healthy_vs_rsv_inf_infant_pbmc_dn	1,00e-05	1,22e-02	Genes down-regulated in comparison of peripheral blood mononuclear cells (PBMC) from healthy donors versus PBMCs from infant with acute RSV infection.
3	gse29617_day3_vs_day7_tiv_flu_vaccine_pbmc_2008_dn	1,00e-05	1,22e-02	Genes down-regulated in comparison of peripheral blood mononuclear cells (PBMC) from TIV influenza vaccinee at day 3 post-vaccination versus those at day 7 post-vaccination.
4	gse34205_rsv_vs_flu_inf_infant_pbmc_up	1,00e-05	1,22e-02	Genes up-regulated in comparison of peripheral blood mononuclear cells (PBMC) from infants with acute RSV infection versus PBMCs from infants with acute influenza infection.
5	gse18281_perimedullary_cortical_region_vs_whole_medulla_thymus_up	3,00e-05	2,92e-02	Genes up-regulated in thymus perimedullary cortical region versus the whole medulla
6	gse22886_tcell_vs_bcell_naive_up	1,20e-04	7,04e-02	Genes up-regulated in comparison of naive CD4 [GeneID = 920] CD8 T cells versus naive B cells.
7	gse6269_healthy_vs_staph_aureus_inf_pbmc_dn	1,20e-04	7,04e-02	Genes down-regulated in comparison of peripheral blood mononuclear cells (PBMC) from patients with acute influenza infection versus PBMC from patients with acute S. aureus infection.
8	gse22886_naive_tcell_vs_monocyte_up	1,30e-04	7,04e-02	Genes up-regulated in comparison of naive CD4 [GeneID = 920] CD8 T cells versus monocytes cultured for 0 days.
9	gse6269_healthy_vs_e_coli_inf_pbmc_up	1,30e-04	7,04e-02	Genes up-regulated in comparison of peripheral blood mononuclear cells (PBMC) from healthy donors versus PBMC from patients with acute E. coli infection.
10	gse10325_cd4_tcell_vs_myeloid_up	1,80e-04	8,77e-02	Genes up-regulated in comparison of healthy CD4 [GeneID = 920] T cells versus healthy myeloid cells.

## Discussion

This explorative analysis has demonstrated a novel and unexpected finding, showing a strong transient increase in significantly differentially expressed genes 3 years or more after breast cancer diagnosis, mainly among women with metastatic disease who later died. Women with invasive cancer who survived showed a negligible number of differentially expressed genes compared to healthy controls, while women with metastatic cancer who survived, and women with invasive cancer who died, had intermediate gene expression patterns.

To our knowledge, this is the first report on changes in global blood gene expression profiles after breast cancer diagnosis with such a long follow-up time. Compared to healthy women, the changes in gene expression depended on the extent of disease at diagnosis, and on the ultimate outcome of death or survival.

Our use of randomized follow-up time after diagnosis in this study is novel, as the follow-up time was not determined by cancer stage at diagnosis, later clinical changes, or other events (see Methods for further details). This design gave a fairly uniform distribution of samples that ensured unbiased estimates of gene expression over time (Fig 1). We used matched controls to describe normal development of gene expression over the entire study period. The use of a randomized follow-up time for each case gave similar statistical power to detect time-dependent changes, independent of surveillance routines at hospitals or disease development. Almost all breast cancer patients in Norway are treated in national hospitals, free of charge. The treatment is defined by the Directorate of Health together with the National Breast Cancer Group, a nationwide organization of oncologists, pathologists, and surgeons. Treatment is given as standardized treatments or “packages”, with a time guarantee. Invasive breast cancer has a normal treatment schedule of less than one year, while metastatic cancer therapies differ more and last longer. Standardized medical revisits are at 1, 2, 5 and ten year after diagnosis. The large number of differentially expressed genes in the first year after diagnosis likely reflects this treatment regime. Of note, information on individual treatment regimens were not available. However, there was no coinciding pattern of our transient gene expression increase and the time points of diagnoses for, and hence treatment of subsequent metastases or second breast cancers in the study population.

### Validity

The validity of the presented analyses depends both on the epidemiological design and the technical quality of the mRNA analyses. It should be mentioned that this is the first time that the registry information on the development of metastases after primary diagnosis has been made available to the NOWAC study. The exclusion of case-control pairs where the case was diagnosed with cancer during follow-up reduced noise in the analyses. We found no evidence of selection bias in the Post-genome Cohort, as participants had the same cancer incidence in the gene expression observation time and prediction time as women in the overall NOWAC study. There were no statistical differences of major confounders (BMI, smoking status, number of children), but comprehensive data on lifestyle and other factors such as family history of breast cancer was not included in the analyses, and may result in residual confounding of our results.

The Post-genome Cohort biobank offers rare opportunities due to the buffering of RNase in all samples, which ensures the quality of the gene expression profiles over time. Importantly, case-control pairs were always handled together throughout all laboratory procedures, which reduced the potential for differential batch effects between cases and controls.

**Fig 7:**
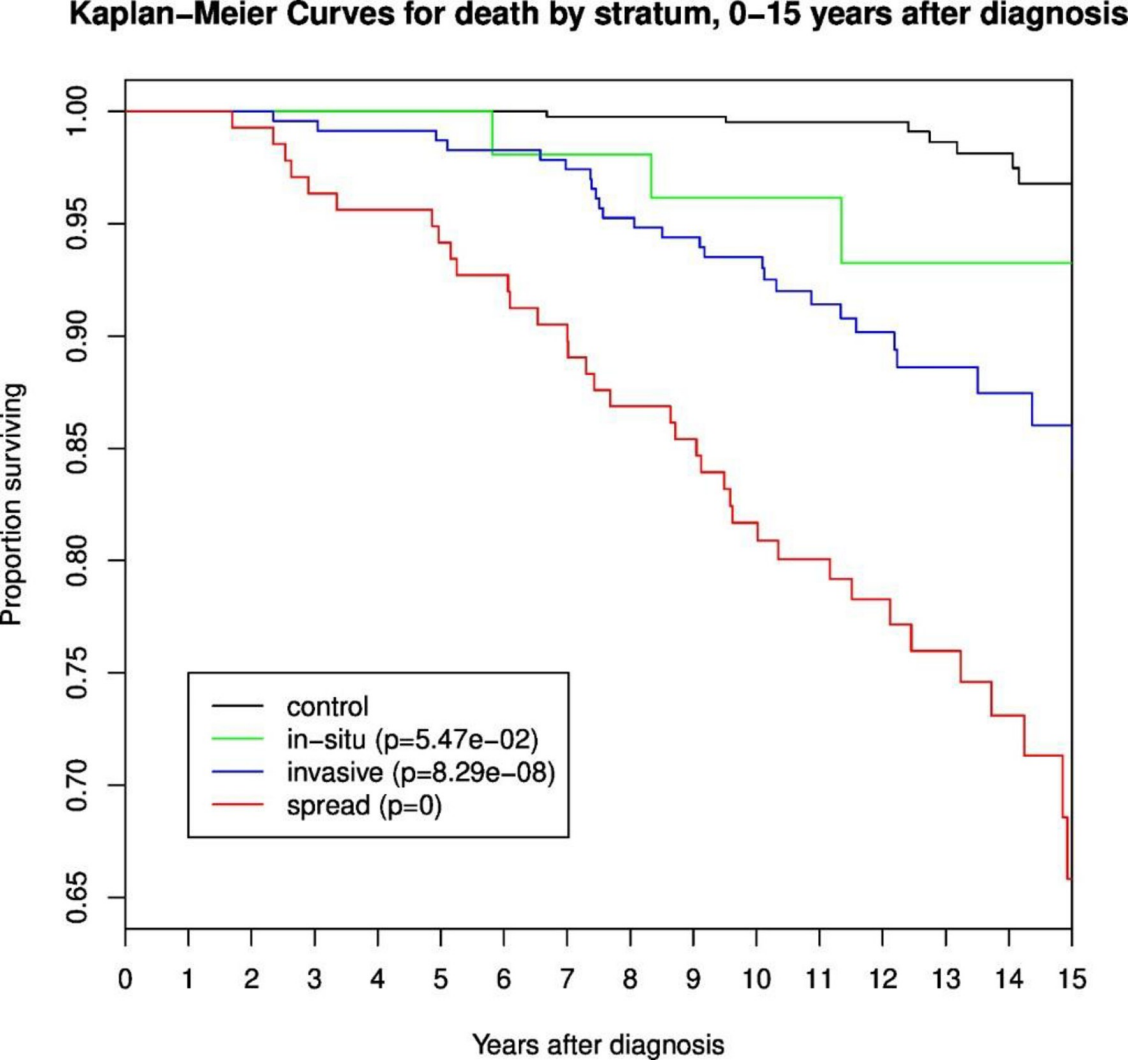
Kaplan-Meier curves for death by stratum. Kaplan-Meier plot for cases and controls in the dataset with up to 15 years of follow-up (metastasis and follow-up time information from the intitial/first breast cancer diagnosis): 52 in situ cases, 232 invasive cases, 137 metastatic cases.

Further strengths of the study are complete follow-up data based on unique national registries and identification numbers, equal access to national health services for all people in Norway, and the good participation rate of the study. Lastly, the analyses were run based on hypotheses stratified on important clinical parameters. The weaknesses of the study include the limited statistical power in stratified analyses and the lack of repeated samples. The study is too small for statistical analyses of additive or multiplicative models. Unfortunately, we did not have information on the dates for recurring breast cancer or metastases in those who were diagnosed with metastatic disease as their primary diagnosis. However, the Kaplan-Meier plot in Fig 7 shows that there is an even distribution of deaths during the 15 years of follow-up. This indicates that the recurrence of disease is evenly distributed in time, with no apparent accumulation of diagnostic events at around four years after the primary diagnosis, which is where our *second transient increase* in the number of differentially expressed genes occurs. This is assessment is also backed by the distribution of time points for the diagnosis of metastasis and a second breast cancer among all cases. Still, we cannot rule out the potential effects of treatment linked to the diagnosis of recurrence of disease.

After 15 years of follow-up, survival among controls was still as high as 97%. The observed survival rates for women with metastatic cancer was almost the same as the relative survival rate. Thus, other causes of death were not considered significant in the metastatic cancer subgroup.

### Biological hypotheses linked to the second transient increase in metastatic cases who died during follow-up

The main aim of this work was to statistically detect and describe the trajectories of gene expression patterns after breast cancer diagnosis, however, some biological interpretation of the findings should be attempted. Our findings regarding the trajectories are novel, and understanding the clinical and biological implications of the second transient increase is challenging. Importantly, our findings are generated from immune cells in peripheral blood. We have previously described that blood-derived transcriptomic profiles differ between breast cancer cases and controls up to 8 years before diagnosis [2, 4], and that there are differences in pre-diagnostic profiles up to two years before diagnosis also when comparing cases with and without metastasis [7]. Finally, we have previously shown that there was no strong correlation between genes expressed in cancer tissue compared to blood samples taken at the same time, except for some highly immunogenetic breast cancer subgroups [8]. Together, these findings indicate that the immunological changes that occur during the carcinogenic process are dynamic reactions to, or interactions with, the growing cancer, as opposed to a mirror of the tumor gene expression.

Among our significantly enriched pathways were heme metabolism, the process of conversion of heme to billiverdin by heme oxygenase-1 (HO-1) and further to bilirubin by billiverdin reductase 1A (BLVR-A). It has recently been established that billiverdin and billirubin are strong anti-oxidants, and that the associated metabolic enzymes are regulators of inflammatory processes [9]. The B isoform of BLVR was among our significantly differentially expressed genes, and this isoform is known to be expressed in immune cells. Aberrant expression levels of BLVR may contribute to pathologies involving the immune system, including cancer [9].

Billirubin has been implicated in control of T-cell function [9], and several of our differentially expressed genes are represented in T cell-related gene sets. T cells play central roles in cancer immune editing mechanisms, the complex interaction between tumor cells and the immune system. The main steps of immune editing are elimination of tumor cells, equilibrium, and finally escape of the cancer cells from the immune system. T regulatory cells at the primary tumor site may promote tumor progression by limiting the immune response towards the tumor [10]. Subsequently, cytotoxic T effector cells are inhibited in their cytolytic activity. A well-described hypothesis for the interaction between disseminated cancer cells and immune system cells in the circulation, states that disseminating cells are rapidly killed outside the protective environment of the primary tumor. Only rarely are they able to establish a new, immunosuppressive microenvironment at distal sites, where the metastatic cancer is allowed to grow [10, 11]. What factors tip the balance between survival and death of disseminated cancer cells is not well described, and it involves both acquired intrinsic characteristics of the cancer cells, and characteristics of the immune cells they encounter. Studies have shown that depletion of cytotoxic T cells enable metastatic tumors to grow, but it is not clear if this depletion is a passive process over time, or actively induced by the tumor cells [10]. We hypothesize that the second transient increase in differentially expressed genes, is a reflection of the final stage of immune editing, the escape of the metastatic cancer cells from the immune system. Although more than 1600 blood cell genes were differentially expressed in the transient increase of our data, the progressing cancers continued to advance, and, ultimately, escaped. Importantly, the Venn diagram (Fig 6) clearly shows the highly different gene expression in each strata, demonstrating a potential to predict the outcome of death in cases with metastatic cancers.

The prevailing view of cancer outlines a somatic selection hypothesis, focusing on stochastic, oncogenic mutations that lead to clones of cells that have higher growth fitness than normal cells [12]. When taking into account the complex interactions between the immune system and cancer, as well as life events or exposures that may influence the immune system, we derive at a more dynamic model of carcinogenesis and metastasis. For decades, epidemiologists have studied the risk factors for breast cancer, showing that changes in exposures over time are reflected in changing risks; studies have pointed to exposure to carcinogens as the driving force behind these changes. However, at the same time, epidemiologists had little or no opportunities to study the involvement of the immune system. The lack of quantification of the immune system as a significant contributor to carcinogenesis, has made the epidemiological understanding of the risks somewhat unidimensional. For example, breast cancer is mainly driven by hormonal exposures. However, in a previous analysis of the NOWAC Post-genome Cohort, it was proposed that each pregnancy offered protection against breast cancer through pregnancy-related changes in the immune system, due to the status of the fetus as a semi-allograft [13]. Increasing parity could explain reduced breast cancer risk because it can potentially lead to more immunologically competent memory cell clones that can support the immune system. In sum, our findings demonstrate the insight given by the systems epidemiology approach [14].

## Conclusion

The strong transient functional signals in the blood of women with metastatic cancers who died during follow-up, could offer the potential for further development into a prognostic marker of metastatic breast cancer outcomes. The results add to our understanding of the last stage of carcinogenesis as a balance between the driving forces of exposures and tumor mutations, and the defense of the body through the immune system.

## Material and methods

### The Norwegian women and cancer study and post-genome cohort

The NOWAC study has a prospective design, with recruitment of 172 000 women randomly sampled from the National Population Register in Norway starting in 1991 [15]. These women received a letter of invitation and a questionnaire, and additional questionnaires were sent out at varying intervals. All participating women were followed up through linkage to national registries based on the unique national identification number assigned to all residents of Norway.

In 2002–2005, NOWAC participants were invited to participate in a sub-cohort: the NOWAC Post-genome Cohort [6]. The main purpose of this cohort was to establish a biobank suitable for analyses of functional genomics, in particular transcriptomics. Random samples of NOWAC participants were drawn in weekly batches of 500, until 50,000 women had been recruited into the Post-genome Cohort. These women completed another questionnaire and were asked to send a blood sample. Whole blood samples were collected by a general practitioner or at a health care institution, using the PAXgene Blood RNA collection kit [Preanalytix/Qiagen, Hombrechtikon, Switzerland), and were sent to the Institute of Community Medicine at UiT in Tromsø by post. The PAXgene Blood RNA collection kit contains a buffer that lyses the blood cells and preserves the mRNA profile of the sample, allowing for long-term frozen storage and optimizing the sensitivity of analyses.

The present analysis used a subsample of 31,101 women in the NOWAC Post-genome Cohort who had completed a questionnaire in 1996–1998 and were still alive when blood sampling started in 2002.

### Identification of breast cancer cases and controls

Eligible Post-genome Cohort participants who were diagnosed with incident breast cancer were identified through linkage to the Cancer Registry of Norway. A first linkage identified 445 incident cases of breast cancer diagnosed between the first questionnaire and blood sample collection. Each of these cases was assigned a matched control at random from the Post-genome Cohort with the same birth year and same weekly batch of 500 invited women. Six case-control pairs were excluded due to outliers from the preprocessing of the gene expression datasets [16].

A second linkage to the Cancer Registry of Norway in 2017 revealed 10 controls diagnosed with cancer before 2010, thus they, and their corresponding cases, were removed. Overall survival rates from diagnosis till end of follow-up included 429 breast cancer patients and confirmed the expected differences according to stage through 15 years (Fig 7). Women with *in situ* breast cancer had a non-significant lower survival than controls.

The second linkage also identified seven breast cancer cases with another incident cancer diagnosis, and seven cases with unknown metastasis status, these were also excluded. This left a final study sample of 415 breast cancer cases. Among these, information on new diagnoses of metastasis and new diagnosis of a second breast cancer during follow-up was also extracted.

### Epidemiological design

The present study has a unique prospective design (Fig 1). Women were followed from the questionnaire in 1996–1998 until death or end of follow-up on 31 December 2014, hereafter referred to as the full study period. However, this period was broken up into three specific sections. The time between the first questionnaire and blood sampling (the waiting time); the time between breast cancer diagnosis and blood sampling; (the gene expression observation time); and the time between blood donation and death or the end of follow-up (prediction time, Fig 1). As the second linkage identified six women with a diagnosis of metastasis after the primary breast cancer diagnosis and 10 women with a second breast cancer during the time of blood sample collection, the gene expression observation time for these women was moved from the primary breast cancer diagnosis to the time of the latest event (i.e., metastasis or second breast cancer) in order to update clinical stage closer to blood sample collection.

The gene expression observation time covered up to 8 years after breast cancer diagnosis. Since NOWAC Post-genome Cohort participants were sampled at random from the overall NOWAC study regardless of disease status, the gene expression observation time also became random and independent of other aspects of the disease. Moreover, this randomization gave an almost uniform distribution of gene expression observation times and measurements (green circles in Fig 1).

We found no selection bias when comparing breast cancer incidence between women in the overall NOWAC study and those in the NOWAC Post-genome Cohort (1.47% versus 1.65%, p = 0.47). Similarly, during the prediction time, women in the NOWAC Post-genome Cohort had a breast cancer incidence of 2.04% compared to 2.00% in the overall NOWAC study.

### Laboratory procedures

All laboratory services were provided by the Genomics Core Facility, Norwegian University of Science and Technology, Trondheim, Norway. To control for technical variability such as different batches of reagents and kits, day-to-day variations, microarray production batches, and effects related to different laboratory operators, each case-control pair was kept together throughout all extraction, amplification, and hybridization procedures. Total RNA extraction was performed using the PAXgene Blood RNA kit (Preanalytix/Qiagen, Hombrechtikon, Switzerland) according to the manufacturer’s instructions. RNA quality and purity were assessed using the NanoDrop ND 8000 spectrophotometer (ThermoFisher Scientific, Wilmington, DE, USA) and Agilent bioanalyzer (Agilent Technologies, Palo Alto, CA, USA). RNA amplification was performed on 96-wells plates using 300 ng of total RNA and the Illumina TotalPrep-96 RNA Amplification Kit (Ambio, Inc., Austin, TX, USA). The mRNA amplification procedure consisted of using a oligo(dT) primer for reverse transcription with a T7 promoter-specific ArrayScript reverse transcriptase, followed by a second-strand synthesis. *In vitro* transcription with T7 RNA polymerase using a biotin-NTP mix produced biotinylated cRNA copies of each mRNA in the sample. All case-control pairs were run on the Illumina HumanHT-12 version 4 bead chip array (Illumina, San Diego, California, USA). Outliers were detected using the R-package Nowaclean [16], based on visual examination of dendrograms, principal component analysis plots and density plots. Individuals that were considered borderline outliers were excluded if their laboratory quality measures were below given thresholds (RIN value <7, 260/280 ratio <2, 260/230 ratio <1.7, and 50 < RNA < 500). Ineligible were six aforementioned case-control pairs where either the case or the control was a technical outlier. All laboratory work was done consecutively for all case-control pairs in 2012.

#### Preprocessing of microarray data

The initial dataset consisted of 445 case-control pairs and 47,285 probes. We excluded 38 probes related to genes in the human leukocyte antigen system, six case-control pairs with outliers, and 10 new incident breast cancer cases, as described above. The resulting dataset with 429 case-control pairs was then background corrected using negative control probes, log_2_ transformed using a variance stabilizing technique [17], and quantile normalized. We retained probes present in at least 70% of women. A probe was defined as present for an individual if its detection p‐value was less than 0.05 for that individual. If a gene was represented with more than one probe, the average expression of the probes was used as the expression value for the gene, resulting in a dataset with 8400 genes. The probes were translated to genes using the lumiHumanIDMapping database [18]. Finally, the differences of the log_2_ gene expression levels for each case-control pair were computed and used in the statistical analyses.

### Statistical methods

#### Identifying differentially expressed genes using a moving window in time

We identified significantly differentially expressed genes using the Bioconductor R-package limma (linear models for microarrays, [19]). In all limma analyses, the response is the difference in log_2_ gene expression between the case and the control of a case-control pair.

To examine how the number of significantly differentially expressed genes varies with time after breast cancer diagnosis, we divided the time after diagnosis into time periods using a moving window in time. For each time period, we identified the number of significantly differentially expressed genes using limma (comparing cases and controls, FDR 5%). The overlapping time periods are defined as follows: let *T* be the number of case-control pairs after diagnosis, and let *t*_1_≤*t*_2_≤⋯≤*t_T_* be the time to diagnosis for these T pairs. The *T*−*S*+1 time periods are defined as the intervals [*t*_1_, *t_S_*], [*t*_2_, *t*_*S*+1_], …, [*t*_*T−S*+1_, *t_T_*] where S≈T4.

To illustrate how the number of identified genes varied with time, we plotted the number of genes for each time period against time since diagnosis, which was defined as the mean time since diagnosis for the cases in that time period. We smoothed the curve using a mean filter with window size 9 and plotted a green circle for every 10th case-control pair.

#### Biological interpretation

To explore significant pathways among the differentially expressed genes in the metastatic cases who died during follow-up, we took two approaches. First, a 2x2Fisher’s test was used on the 1627 differentially expressed genes, as well as a simple overlap analysis from Broad Institute’s Molecular Signatures Database (MSigDB) v.7.0 (http://software.broadinstitute.org/gsea/msigdb, [20]) on the top genes presented in the heat map. Secondly, the fgsea enrichment analysis was employed.

First, because the present analysis does not provide a standard gene expression matrix, well-known functional class scoring methods like GSEA, mroast or similar, could not be used without modifications. Hence, a 2x2 Fisher’s test was used to compare the 1627 genes identified in those metastatic cases who died, to the immunological gene sets in the C7 collection of MSigDB. The test was adjusted for multiple testing using the false discovery rate (FDR, [21]), to control the type 1 error rate (mistakenly rejecting a null hypothesis when it is in fact true). Focusing on the top 50 identified genes, an overrepresentation analysis based on the Fisher’s test and provided by Broad Institute web page [20], gave clues on overlaps compared to both the C7 immunological gene set collection, as well as the H hallmarks collection [22] in MSigDB. The latter gene set collection describes gene sets included in well-defined biological states or processes.

For the second approach, we used the Bioconductor R-package fgsea (fast gene set enrichment analysis, [23]). This method requires a pre-ranking of the genes. For each gene *i* we compute the rank value *V_i_* as *V_i_* = *S_i_−k_i_p_i_*, where *S_i_* is the number of time periods where the gene is significant. If *S_i_*>0, then *p_i_* is the median p-value in the *S_i_* intervals with significant FDR q-value (FDR 5%) and $k_{i}=0.8/{max}_{j\;for\;S_{j}>0}\{p_{j}\}$. If *S_i_* = 0, then *p_i_* is the minimum p-value in all the intervals and $k_{i}=0.8/{max}_{j\;for\;S_{j}=0}\{p_{j}\}$. This gives a complete ranking of the genes, primarily based on the number of time periods with significant FDR q-values. MSigDB collections H hallmark gene sets, and C7 immunological gene sets were used as input [20].

### Ethical issues

The NOWAC study was approved by the Norwegian Data Inspectorate and recommended by the Regional Ethical Committee of Northern Norway (REC North). The linkages of the NOWAC database to national registries such as the Cancer Registry of Norway and registries on death and emigration have also been approved, and the women were informed about these linkages. Furthermore, the collection and storing of human biological material was approved by the REC North in accordance with the Norwegian Biobank Act. Information on cancer cases were retrieved from the Cancer Registry of Norway. The linkages between Cancer Registry data and NOWAC study participants were performed at Statistics Norway, and the dataset was fully anonymized before it was made available to the authors. The Cancer Registry of Norway is established by the Norwegian Health Registry Act, and is required to, without consent, contain records of personal, administrative, medical, diagnostic, treatment, and residual cancer data. The Cancer Registry is updated every year, and records until the end of 2016 were used in this study. The Norwegian Data Protection Authority gave NOWAC exemption from the duty of confidentiality and permission to handle personal data (Datatilsynet, ref. 07/00030-2/cbr).

## Supporting information

S1 Appendix Descriptive characteristics of breast cancer cases and healthy controls. (DOCX)

S2 Appendix List of 1627 differentially expressed genes in blood samples of breast cancer patients with metastasis who died during follow-up, compared to healthy controls. We performed limma analyses with a moving window in time to identify time periods after breast cancer diagnosis where cases and controls had different gene expression profiles. Significant genes were defined as genes that were significant for at least one time period out of a total of 22 periods. If any genes reached significance in an identical number of periods, the median of the p-values was calculated, and the genes were sorted by this median. The list is sorted by the number of time periods where each gene reached significance, hence the top of the list are the genes that were significant in the highest number of periods.
(XLSX)
