# Supplementary material for: Global blood gene expression profiles following a breast cancer diagnosis—Clinical follow-up in the NOWAC post-genome cohort
Source: PLoS One. 2021 Mar 8;16(3):e0246650. doi: 10.1371/journal.pone.0246650 (PMC7939296; doi:10.1371/journal.pone.0246650)
Supplement: S1 Appendix — (DOCX) [file pone.0246650.s001.docx]

Appendix to Olsen et al. Global blood gene expression profiles following a breast cancer diagnosis – clinical follow-up in the NOWAC Post-genome cohort

**Table: Descriptive statistics for cases and controls**

|  | Cases | Controls | p-value |
| --- | --- | --- | --- |
| N |  |  |  |
| BMI, mean (SD) | 25.9 (4.4) | 25.8 (4.1) | 0.780 |
| Current smokers | 78 | 98 | 0.089 |
| N. children, mean (SD) | 2.2 (1.0) | 2.3 (1.1) | 0.348 |

Information on BMI and smoking were given at the point of blood draw. Information on number of children was extracted from the NOWAC database. Abbreviations: BMI – body mass index, SD – standard deviation.
